# Supplementary material for: Mild photothermal therapy potentiates anti-PD-L1 treatment for immunologically cold tumors via an all-in-one and all-in-control strategy
Source: Nat Commun. 2019 Oct 25;10:4871. doi: 10.1038/s41467-019-12771-9 (PMC6814770; doi:10.1038/s41467-019-12771-9)
Supplement: Supplementary file 1 — Supplementary Information [file 41467_2019_12771_MOESM1_ESM.pdf]

# Supplementary Information

**Mild photothermal therapy potentiates anti-PD-L1 treatment for immunologically cold tumors via an all-in-one and all-in-control strategy**

**Huang et al.**

## Supplementary Figures

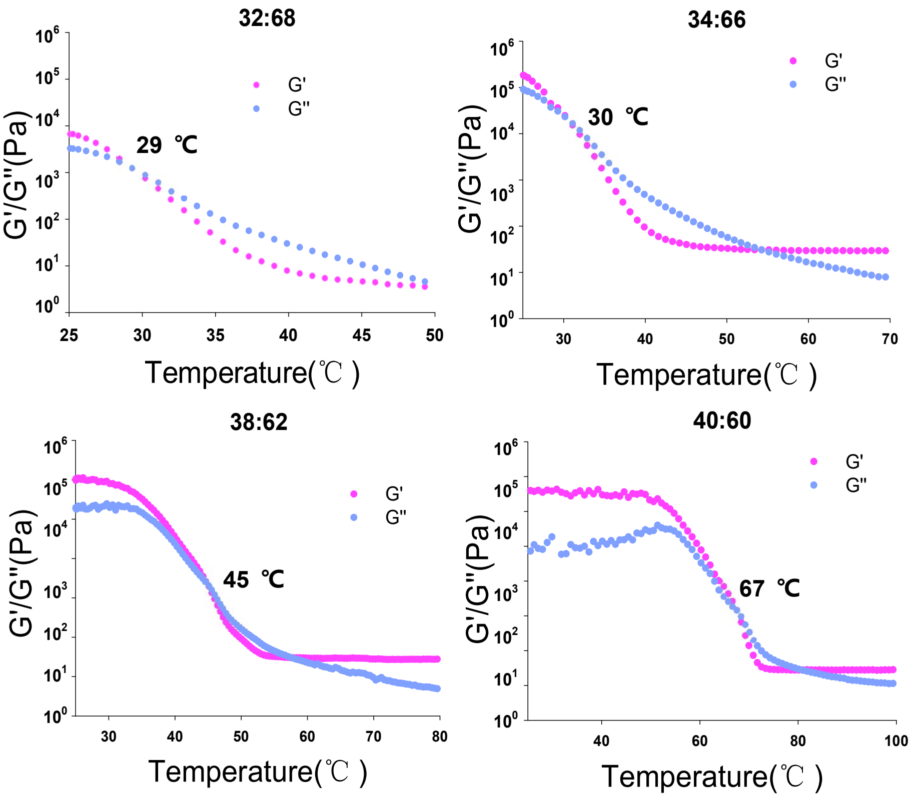


**Supplementary Fig. 1:** The phase transition temperature of different ratio of LG.

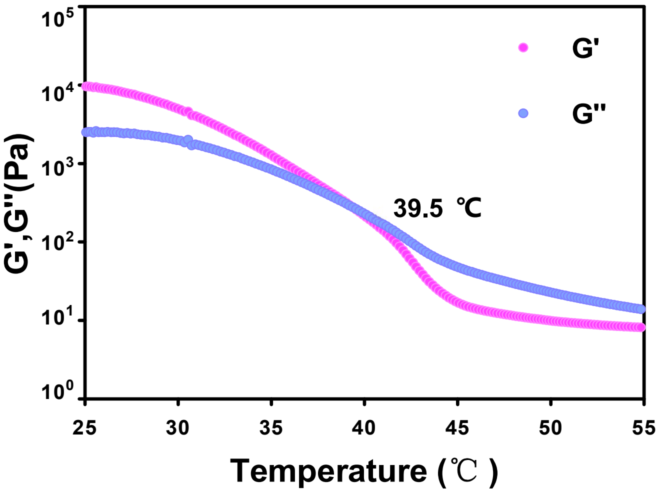


**Supplementary Fig. 2:** Temperature-responsive storage (*G'*) and loss (*G''*) modulus changes of drug-loaded LG (SPC/GDO, 35/65; IR820/IgG, 2 mg $mL^{-1}$). The phase transition temperature of the LG is 39.5 °C.

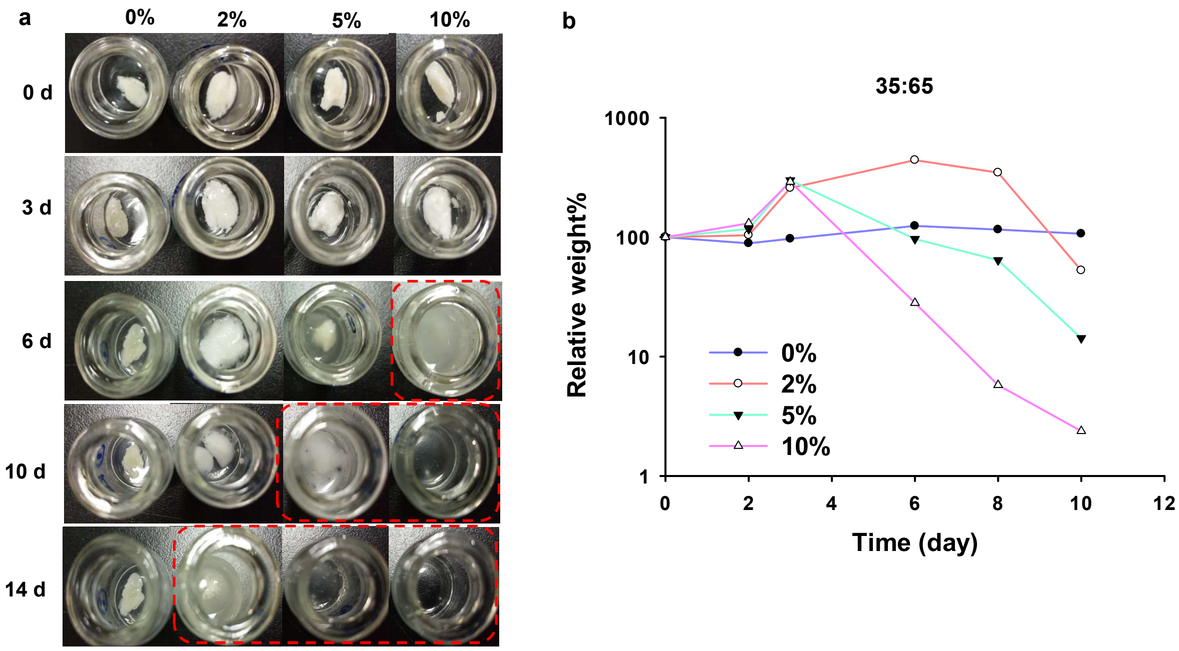


**Supplementary Fig. 3:** *In vitro* degradation behavior (**a**: Morphology changes; **b**: degradation curve) of the gel incubated with 0%, 2%, 5% and 10% lipase over 14 d in PBS (pH 7.4), respectively.

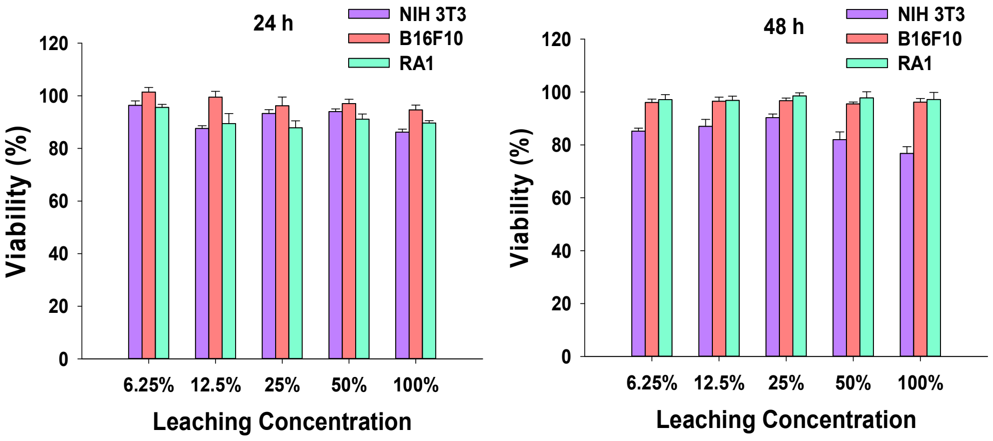


**Supplementary Fig. 4:** The cytotoxicity of LG (SPC/GDO, 35/65) against different cell lines at 24 h and 48 h. Data are presented as mean ± s.e.m. (n = 4 biologically independent samples).

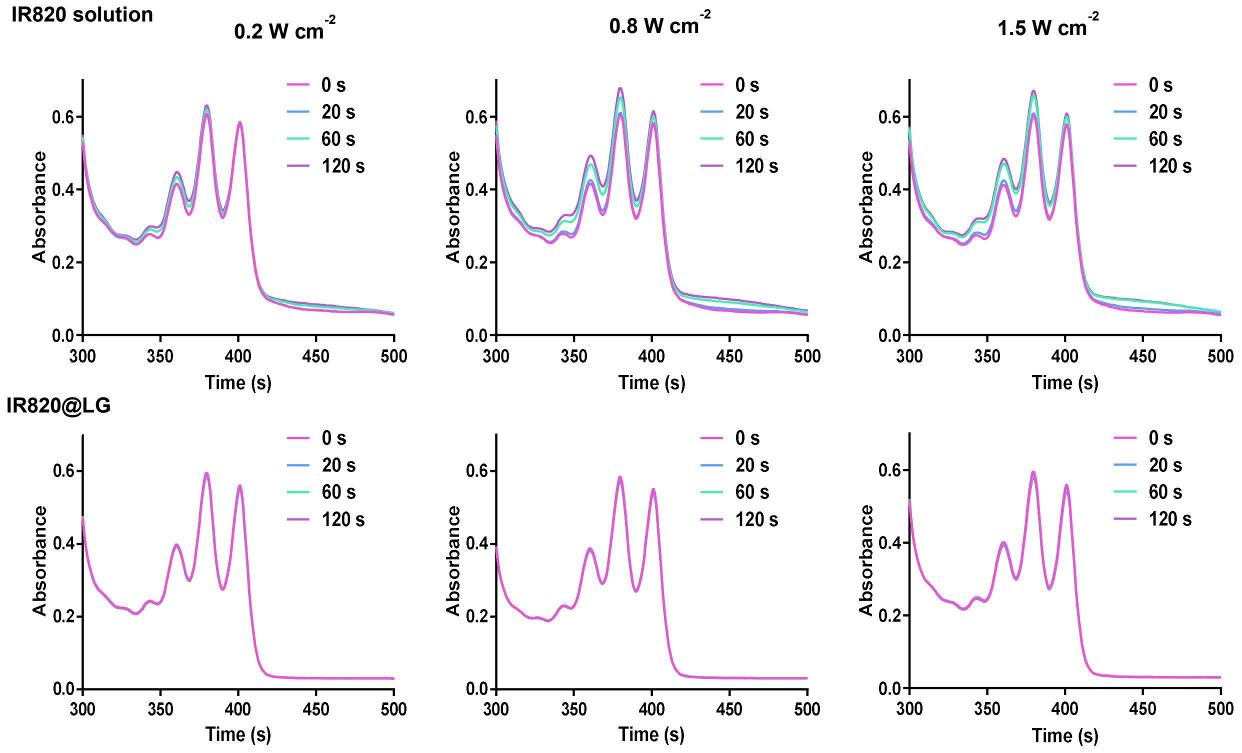


**Supplementary Fig. 5:** The singlet oxygen production analysis with different NIR powers on IR820 solution and IR820@LG using 9,10-anthracenediyl-bis(methylene) dimalonic acid (ABDA) as the singlet oxygen sensor.

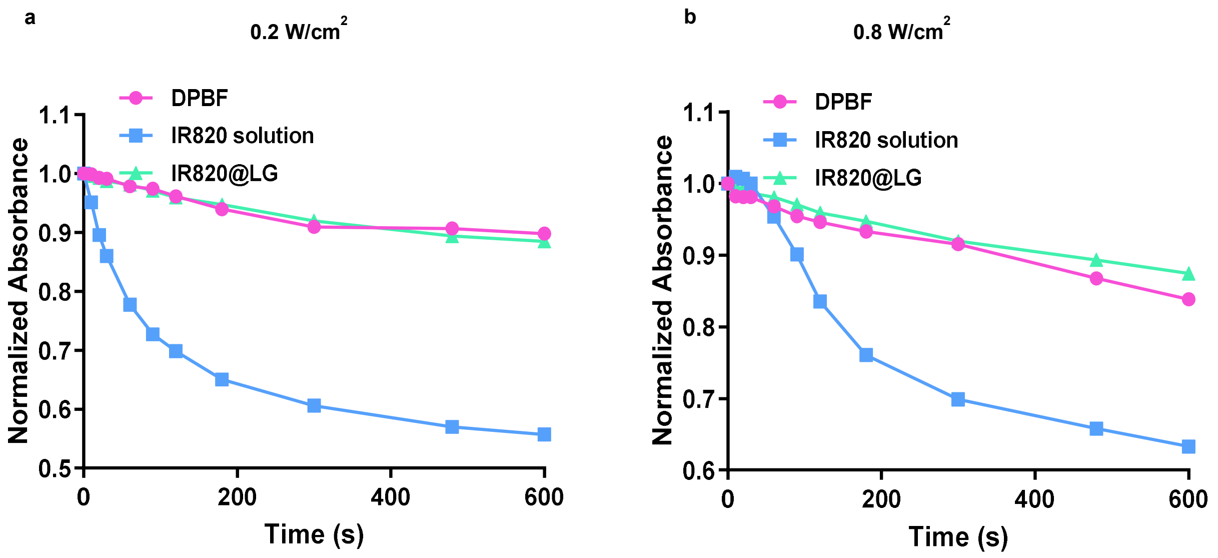


**Supplementary Fig. 6:** The singlet oxygen generation analysis (**a**: 0.2 W cm$^{-2}$, **b**: 0.8 W cm$^{-2}$) by a diphenylisobenzofuran (DPBF) based UV spectroscopic method.

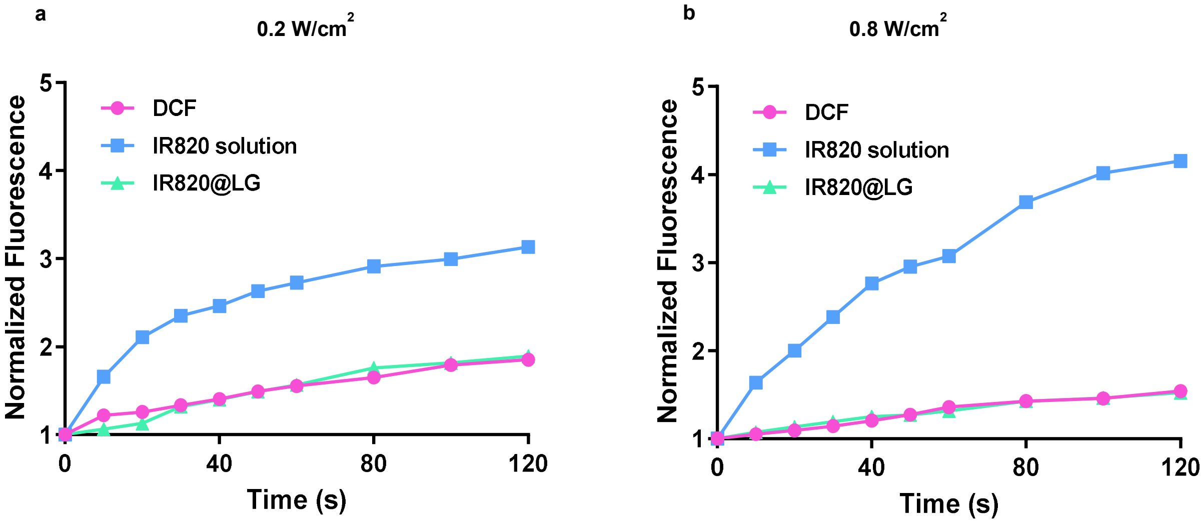


**Supplementary Fig. 7:** The reactive oxygen species (ROS) generation analysis (**a**: 0.2 W cm$^{-2}$, **b**: 0.8 W cm$^{-2}$) by the dichlorodihydrofluorescein diacetate (DCF-DA) based fluorescence spectroscopic method.

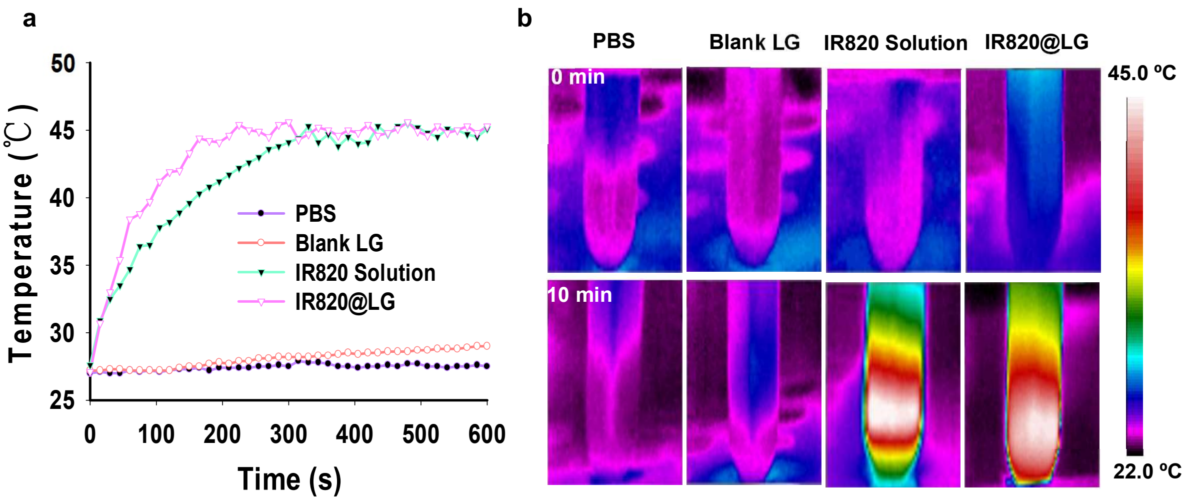


**Supplementary Fig. 8: a)** Temperature increase curves of PBS, blank LG, IR820 solution and IR820@LG with the NIR laser irradiation for 10 min *in vitro*. **b)** *In vitro* infrared thermal images of PBS, blank LG, IR820 solution and IR820@LG before and 10 min after illumination.

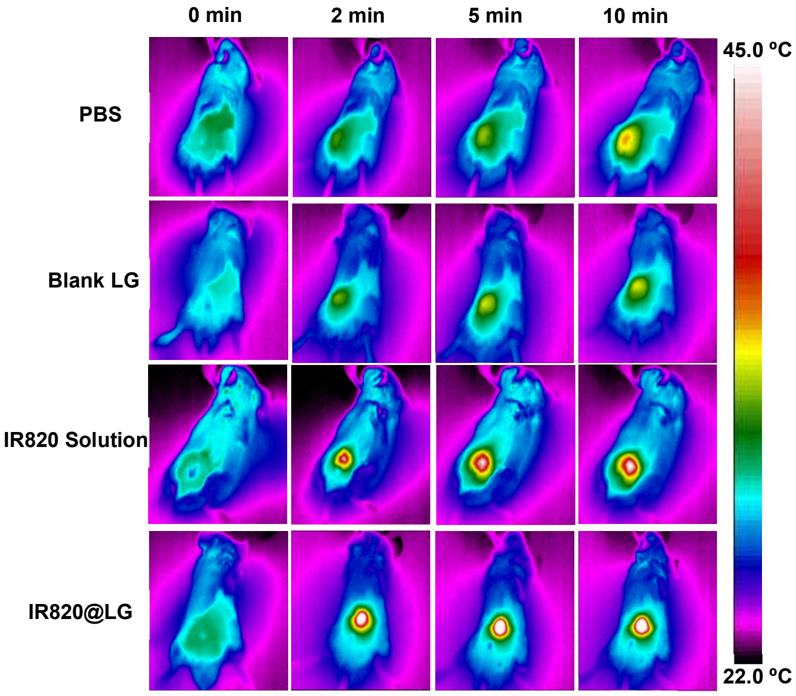


**Supplementary Fig. 9:** In vivo infrared thermal images of the injection sites in normal mice irradiated immediately post injection with PBS, blank LG, IR820 solution and IR820@LG. Images were recorded at 0 min, 2 min, 5 min and 10 min after irradiation.

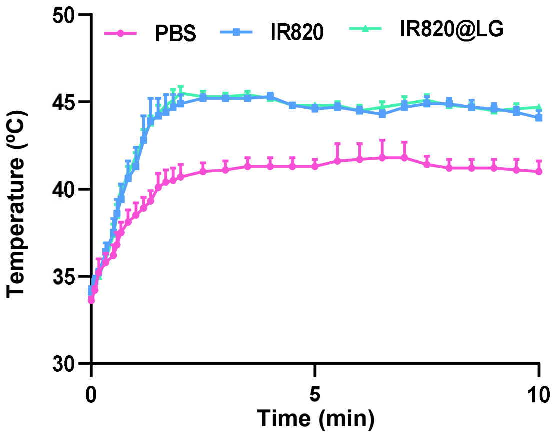


**Supplementary Fig. 10:** Temperature variation curves of the tumor sites in B16F10-bearing mice after intratumor injection with PBS, IR820 solution and IR820@LG, followed by NIR laser irradiation. Data are shown as means ± s.e.m. (n = 3 biologically independent samples).

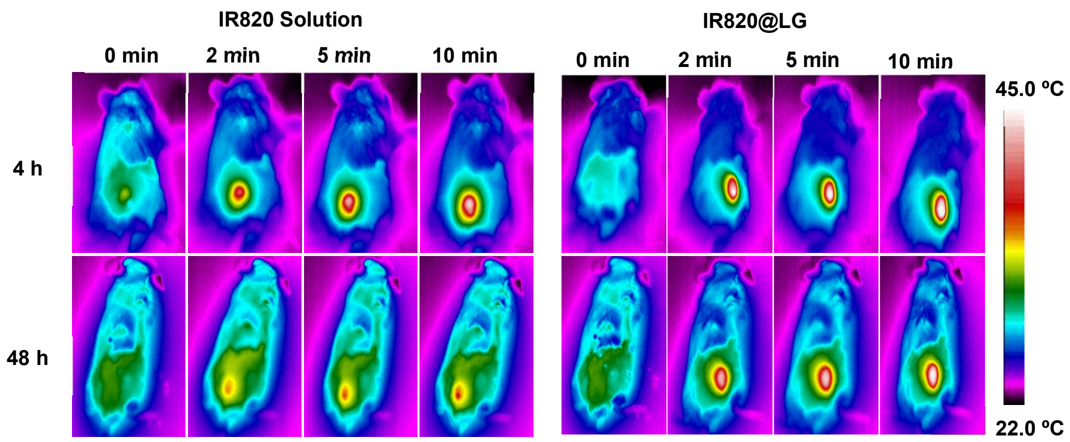


**Supplementary Fig. 11:** In vivo infrared thermal images of the tumor sites in 4T1 tumor-bearing mice irradiated at 4 h and 48 h post intratumor injection with IR820 solution and IR820@LG. Images were recorded at 0 min, 2 min, 5 min and 10 min after irradiation.

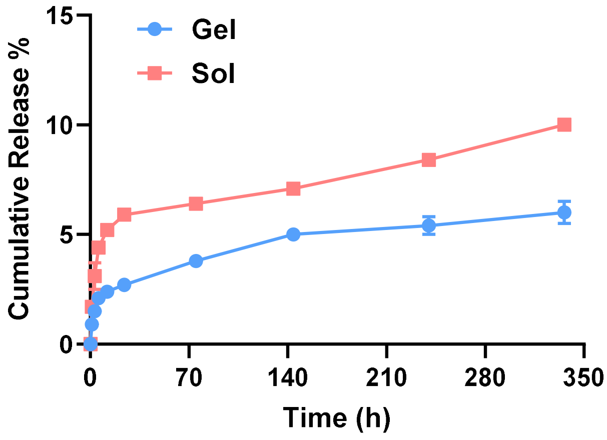


**Supplementary Fig. 12:** Cumulative release profiles of IgG from the sol or gel forms of LG in PBS under different incubation conditions. Data are presented as mean ± s.e.m. (n = 3 independent samples).

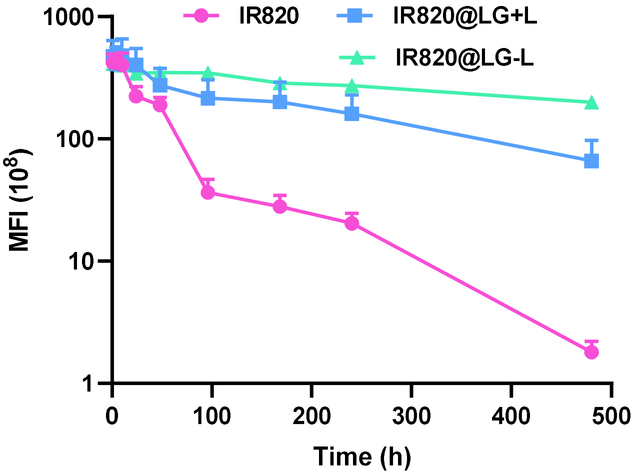


**Supplementary Fig. 13:** Mean fluorescence intensity (MFI) of the intratumor drug retention time of IR820@LG in 4T1-bearing mouse models with 808 nm laser irradiation. Data are shown as means ± s.e.m. (n = 3 biologically independent samples).

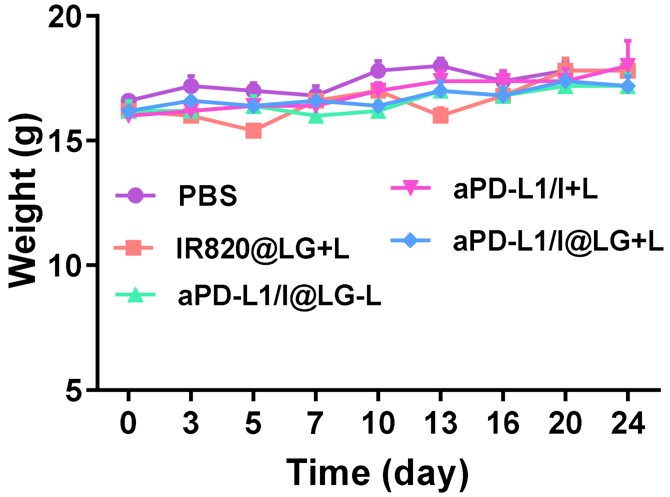


**Supplementary Fig. 14:** The body weight change curve of the 4T1-bearing mouse models. Data are presented as mean ± s.e.m. (n = 5 biologically independent samples).

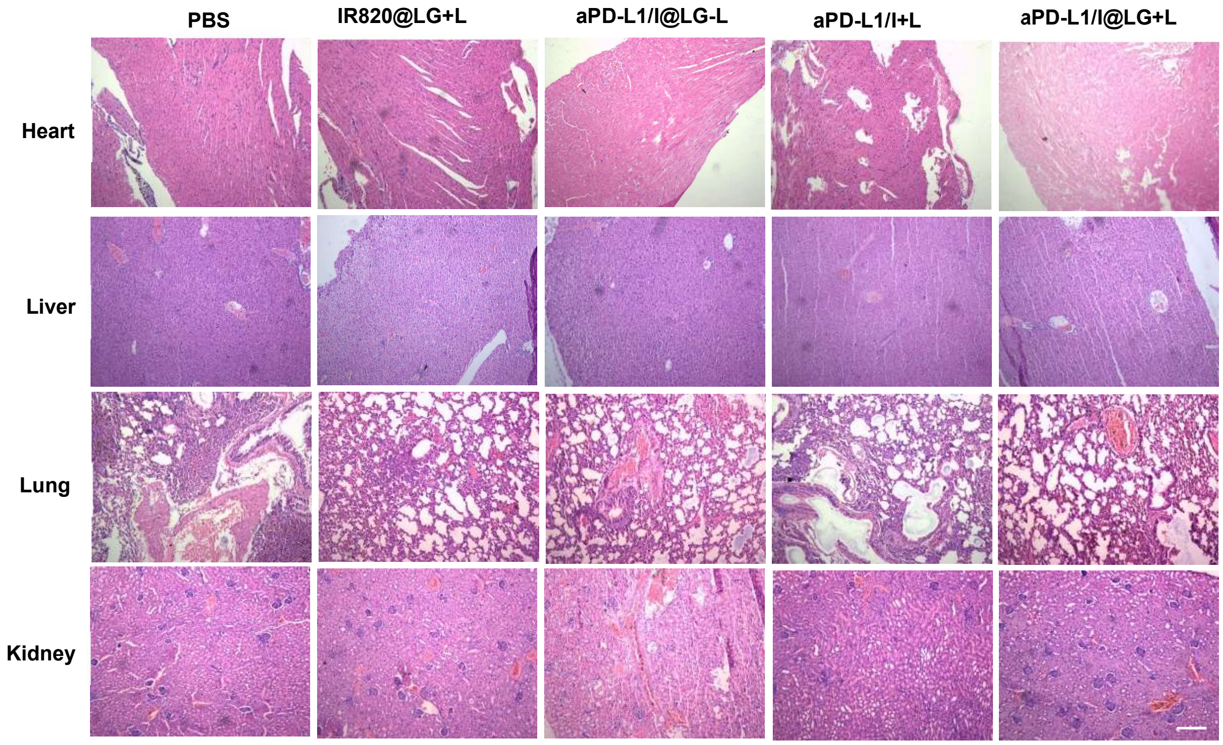


**Supplementary Fig. 15:** Histopathological images of the main organs, containing liver, lung, kidney and heart obtained from the 4T1-bearing mice. The images were measured at a magnification of 100× (scale bar: 200 μm).

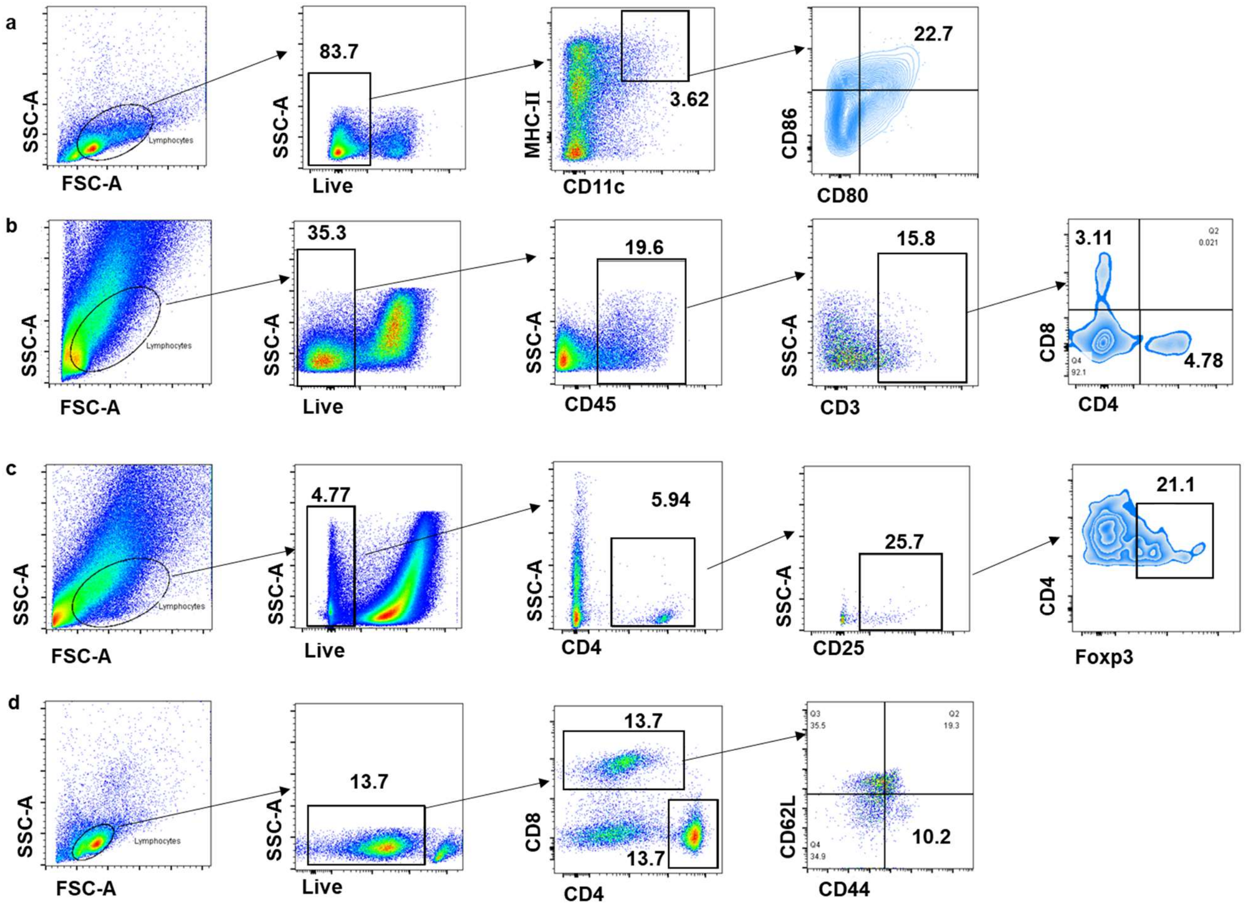


**Supplementary Fig.16.** Gating strategies used for cell sorting. **a)** Gating strategy to sort matured DC (CD11c$^+$CD80$^+$CD86$^+$) cells from Balb/c mice presented on Fig. 5a,b. **b)** Gating strategy to sort CD8$^+$ (CD45$^+$CD3$^+$CD8$^+$) T cells and CD4$^+$ (CD45$^+$CD3$^+$CD4$^+$) T cells from Balb/c mice presented on Fig. 5c,d. **c)** Gating strategy to sort Treg (CD4$^+$CD25$^+$ Foxp3$^+$) cells from Balb/c mice presented on Fig. 5e,f. **d)** Gating strategy to sort T$_{EM}$ (CD11b$^+$CD8$^+$CD44$^+$CD62L$^-$) cells from Balb/c mice presented on Fig. 7e,f.

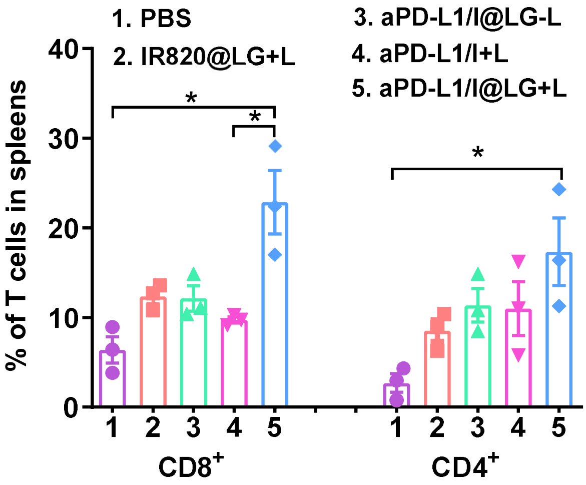


**Supplementary Fig. 17:** Flow cytometric examination of the intratumor infiltration of $CD4^+$ and $CD8^+$ T cells in the spleens (gated on $CD3^+$ T cells). Data represent mean ± s.e.m. (n = 3 biologically independent samples). The comparison of two groups was followed by unpaired student's t-test (two-tailed). *$P < 0.05$.

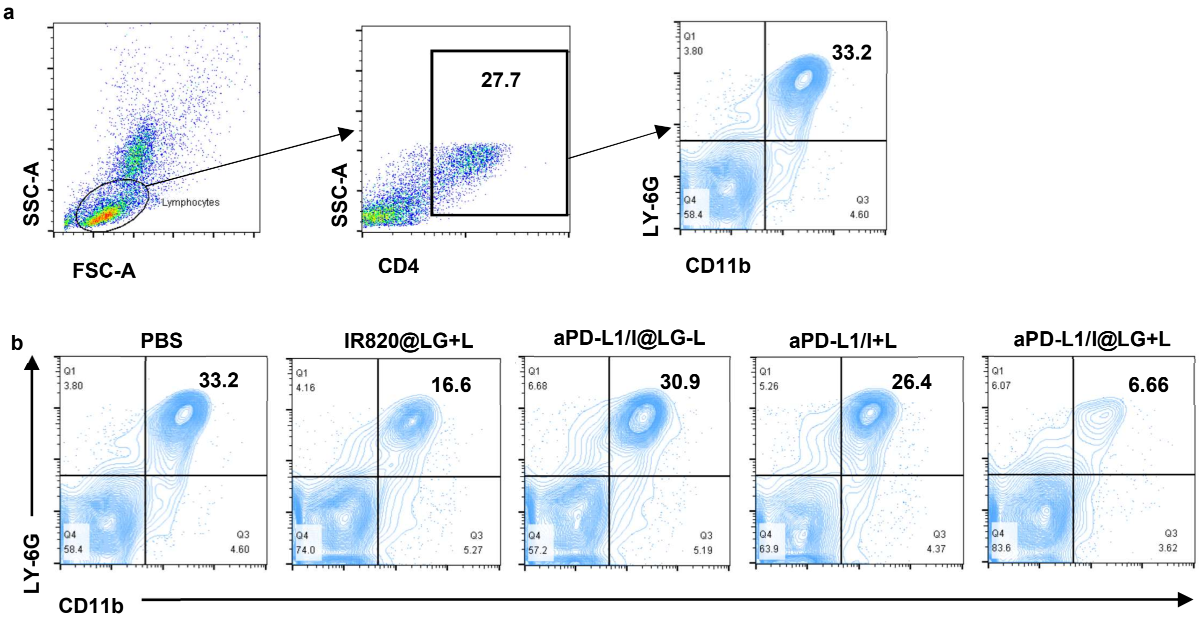


**Supplementary Fig. 18: a)** Gating strategy to sort MDSC(CD4$^+$CD11b$^+$LY-6G$^+$) cells from Balb/c mice presented on Fig. 5g,h and Supplementary Fig.18b. **b)** Representative flow cytometry plots showing the frequency of MDSC in the primary tumors after different treatments examined on the 8$^{th}$ day after treatment.

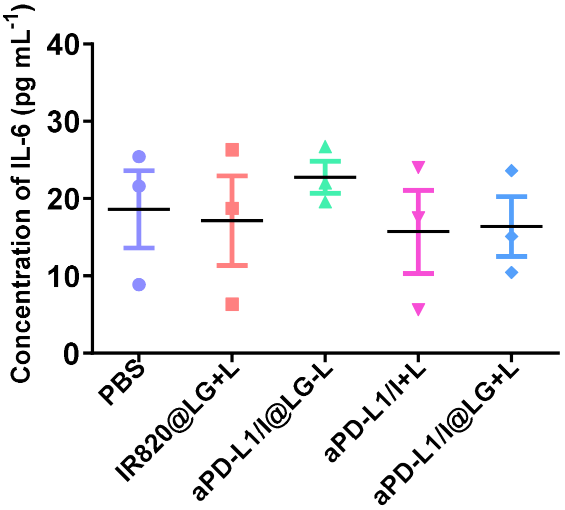


**Supplementary Fig. 19:** Concentration of the IL-6 in plasma on the 8$^{th}$ day after treatment on 4T1 tumor-bearing mice. Data represent mean ± s.e.m. (n = 3 biologically independent samples).

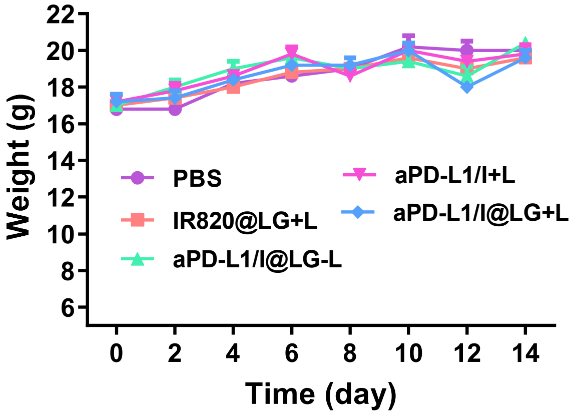


**Supplementary Fig. 20:** The body weight change curves of B16F10-bearing mice. Data are presented as mean ± s.e.m. (n = 5 biologically independent samples).
