# Supplementary material for: Mild photothermal therapy potentiates anti-PD-L1 treatment for immunologically cold tumors via an all-in-one and all-in-control strategy
Source: Nat Commun. 2019 Oct 25;10:4871. doi: 10.1038/s41467-019-12771-9 (PMC6814770; doi:10.1038/s41467-019-12771-9)
Supplement: Supplementary file 3 — Reporting Summary [file 41467_2019_12771_MOESM3_ESM.pdf]

natureresearch

Corresponding author(s): Jiasheng Tu, Liang Luo, Chunmeng Sun

Last updated by author(s): Aug 21, 2019

Reporting Summary

Nature Research wishes to improve the reproducibility of the work that we publish. This form provides structure for consistency and transparency in reporting. For further information on Nature Research policies, see Authors & Referees and the Editorial Policy Checklist .

Statistics

For all statistical analyses, confirm that the following items are present in the figure legend, table legend, main text, or Methods section.

n/a	Confirmed	
☐	☒	The exact sample size (*n*) for each experimental group/condition, given as a discrete number and unit of measurement
☐	☒	A statement on whether measurements were taken from distinct samples or whether the same sample was measured repeatedly
☐	☒	The statistical test(s) used AND whether they are one- or two-sided<br>*Only common tests should be described solely by name; describe more complex techniques in the Methods section.*
☐	☒	A description of all covariates tested
☐	☒	A description of any assumptions or corrections, such as tests of normality and adjustment for multiple comparisons
☐	☒	A full description of the statistical parameters including central tendency (e.g. means) or other basic estimates (e.g. regression coefficient) AND variation (e.g. standard deviation) or associated estimates of uncertainty (e.g. confidence intervals)
☒	☐	For null hypothesis testing, the test statistic (e.g. *F*, *t*, *r*) with confidence intervals, effect sizes, degrees of freedom and *P* value noted<br>*Give P values as exact values whenever suitable.*
☒	☐	For Bayesian analysis, information on the choice of priors and Markov chain Monte Carlo settings
☒	☐	For hierarchical and complex designs, identification of the appropriate level for tests and full reporting of outcomes
☒	☐	Estimates of effect sizes (e.g. Cohen's *d*, Pearson's *r*), indicating how they were calculated

Our web collection on statistics for biologists contains articles on many of the points above.

Software and code

Policy information about availability of computer code

Data collection	Data was collected using FLIR E50, IVIS Lumina XR, CLSM Zesis 710, Kinexus Rotational Rheometer Malvern Instrument, BD FASCVerse, ImageJ
Data analysis	Statistical analyses were performed using GraphPad Prism software. FlowJo was used to process all the flow cytometry data. Living Image software was used to process mouse images. FLIR E50 was used to process photothermal images.

For manuscripts utilizing custom algorithms or software that are central to the research but not yet described in published literature, software must be made available to editors/reviewers. We strongly encourage code deposition in a community repository (e.g. GitHub). See the Nature Research guidelines for submitting code & software for further information.

Data

Policy information about availability of data

All manuscripts must include a data availability statement. This statement should provide the following information, where applicable:

- Accession codes, unique identifiers, or web links for publicly available datasets
- A list of figures that have associated raw data
- A description of any restrictions on data availability

Data Availability. The authors declare that all the data supporting the findings of this study are available within the article and its Supplementary Information files or from the corresponding author upon reasonable request.

Field-specific reporting

Please select the one below that is the best fit for your research. If you are not sure, read the appropriate sections before making your selection.

Life sciences study design

All studies must disclose on these points even when the disclosure is negative.

Sample size	Details regarding the sample size of all experiments are provided in figure legends. Sample size were estimated to achieve about 90% power for detection of significant differences in tumor volume between groups based on means and standard deviations in preliminary studies. They were consistent with sample size of previously reported results in other studies.
Data exclusions	No data were excluded from the analyses.
Replication	In vitro experiments were completed in duplicate or triplicate to successfully verify reproducibility. In vivo experiments were completed in triplicate as noted by up to three people to successfully verify reproducibility and the results were pooled as noted.
Randomization	For in vitro test, samples were randomly allocated to corresponding experimental groups. For in vivo test, mice were inoculated tumor at the same time and then randomly assigned to a group for similar average tumor sizes.
Blinding	The investigator was blinded to the group allocation during the tumor size measurement, tissue harvesting and processing.

Reporting for specific materials, systems and methods

We require information from authors about some types of materials, experimental systems and methods used in many studies. Here, indicate whether each material, system or method listed is relevant to your study. If you are not sure if a list item applies to your research, read the appropriate section before selecting a response.

Materials & experimental systems

n/a	Involved in the study	
☐	☒	Antibodies
☐	☒	Eukaryotic cell lines
☒	☐	Palaeontology
☐	☒	Animals and other organisms
☒	☐	Human research participants
☒	☐	Clinical data

Methods

n/a	Involved in the study	
☒	☐	ChIP-seq
☐	☒	Flow cytometry
☒	☐	MRI-based neuroimaging

Antibodies

Antibodies used	aPD-L1 used in vivo purchased from BioLedend Inc. anti-CD3-PerCP-Cy5.5 (catalog no. 551163), anti-CD4-FITC (catalog no. 553046), anti-CD8-PE (catalog no. 553032) , anti-CD4-FITC (catalog no. 553046), anti-CD25-APC (catalog no. 557192), and anti-Foxp3-PE (catalog no. 563101), anti-CD11b-FITC (catalog no. 557396), anti-CD11c-FITC (catalog no. 557400), anti-CD80-PE (catalog no. 560016), anti-CD86-APC (catalog no. 553692), anti-LY-6G/LY/6C-PE (catalog no. 553128), anti-CD3-FITC (catalog no. 553065), anti-CD8-PerCP-Cy5.5 (catalog no. 553030), anti-CD62L-APC (catalog no. 562910), and anti-CD44-PE (catalog no. 559250) were purchased from BD Biosciences.
Validation	The antibodies for flow cytometry were validated by BD Bioscineces, with related data shown on the manufacturer website. The validation of therapeutic antibodies were performed by SDS-PAGE by Bio X Cell, with relevant data presented on the manufacturer website. Additional validation of both therapeutic and flow cytometric antibodies was not performed by the authors.

Eukaryotic cell lines

Policy information about cell lines

Cell line source(s)	The fibroblast NIH 3T3, human blood B lymphocytes RAMOS (RA1), melanoma B16F10 and metastatic murine 4T1 breast cancer cell lines were purchased from the American Type Culture Collection (ATCC).
Authentication	The cell lines were morphologically confirmed according to the information provided by ATCC
Mycoplasma contamination	All cell lines were tested for mycoplasma contamination. Mycoplasma contamination was not found
Commonly misidentified lines (See ICLAC register)	No commonly misidentified cell lines were used.

Animals and other organisms

Policy information about studies involving animals; ARRIVE guidelines recommended for reporting animal research

Laboratory animals	BALB/c mice and C57BL/6 mice (6 to 8 weeks old, 18-20 g) were purchased from the Qinglongshan Farms (Nanjing, China). All animals were bred in the pathogen-free facility with a 12 h light/dark cycle at 20 ± 3 ˚C and had ad libitum access to food and water. Animal protocols were performed under the guidelines for human and responsible use of animals in research set by Huazhong University of Science and Technology and China Pharmaceutical University.
Wild animals	This study did not involve wild animals.
Field-collected samples	This study did not involve field-collected samples.
Ethics oversight	Animal protocols were performed under the guidelines for human and responsible use of animals in research set by Huazhong University of Science and Technology and China Pharmaceutical University.

Note that full information on the approval of the study protocol must also be provided in the manuscript.

Flow Cytometry

Plots

Confirm that:

- [x] The axis labels state the marker and fluorochrome used (e.g. CD4-FITC).
- [x] The axis scales are clearly visible. Include numbers along axes only for bottom left plot of group (a 'group' is an analysis of identical markers).
- [x] All plots are contour plots with outliers or pseudocolor plots.
- [x] A numerical value for number of cells or percentage (with statistics) is provided.

Methodology

Sample preparation	Tumors, spleen and tumor-draining lymph nodes were harvested from sacrificed mice. The tumors and lymph nodes were cut into small pieces and resuspended in collagenase D in DMEM (1 mg/mL). The solutions were incubated for 1 h at 37°C on a shaker (90 rpm) and then filtered through a 70-µm Falcon cell strainer. The supernatant from the digested tumor tissues was collected, centrifuged at 490 × g for 5 min, and resuspended. The spleen was mechanically dissociated and resuspended in DMEM. The suspension was filtered through a 70-µm Falcon cell strainer, centrifuged and resuspended. Erythrocytes were lysed with red blood cell lysis buffer for 5 min at 37°C. Cell suspensions were prepared as described above and then stained with the antibodies. The cells were then washed twice and analysed using flow cytometer.
Instrument	BD FASCVerse Flow Cytometry
Software	Flowjo_V10
Cell population abundance	The cells were extracted by specific gravity separation methods with 70% percoll and 40% percoll.
Gating strategy	Initial cell populations were gated for a live population using FSC and SSC plot of cell only sample. The gate was set to remove cell debris and dead cells (small FSC and SSC) and large clumps or aggregates of cells (large FSC and SSC) and used across all samples. This live population was then further gated as shown in Supplementary Fig. S16,18.

- [x] Tick this box to confirm that a figure exemplifying the gating strategy is provided in the Supplementary Information.
